# Temporal changes in haematocrit following artemisinin-based combination treatments of uncomplicated falciparum malaria in children

**DOI:** 10.1186/s12879-015-1219-y

**Published:** 2015-10-26

**Authors:** Akintunde Sowunmi, Kazeem Akano, Adejumoke I. Ayede, Godwin Ntadom, Bayo Fatunmbi, Temitope Aderoyeje, Elsie O. Adewoye

**Affiliations:** Department of Pharmacology & Therapeutics, University of Ibadan, Ibadan, Nigeria; Institute for Medical Research and Training, University of Ibadan, Ibadan, Nigeria; Department of Paediatrics, University of Ibadan, Ibadan, Nigeria; Federal Ministry of Health, Abuja, Nigeria; World Health Organization, Regional Office for the Western Pacific, Khan Daun Penh, Phnom Penh, Cambodia; Department of Clinical Pharmacology, University College Hospital, Ibadan, Nigeria; Department of Physiology, University of Ibadan, Ibadan, Nigeria

**Keywords:** Malaria, Artemisinin-based combination treatments, Fall in haematocrit, Children, Nigeria

## Abstract

**Background:**

Artemisinin-based combination treatments (ACTs) or intravenous artesunate are used in over 100 countries for uncomplicated or severe falciparum malaria. Although intravenous artesunate may cause delayed haemolytic anaemia, there is little evaluation of the temporal changes in haematocrit following ACTs.

**Methods:**

Clinical and parasitological parameters were measured before and following treatment of uncomplicated falciparum malaria in children with artesunate-amodiaquine (AA) or artemether-lumefantrine (AL) over 6-weeks. Changes in haematocrit were characterized in individual patients based on a haematocrit <30 % or ≥30 % before and following treatment. Kinetics of the deficit in haematocrit from <30 % until attainment of ≥30 % were estimated by a non-compartment model.

**Results:**

In 248 of 1180 children eligible for evaluation, common temporal patterns were: no change or increase in haematocrit from ≥ 30 % [50 % of patients], haematocrit >30 % at presentation declining to <30 % within 2 weeks (early monophasic fall) [19 % of patients], and haematocrit <30 % at presentation increasing to ≥ 30 % [23 % of patients]. Haematocrit >30 % at presentation declining to <30 %, 3–5 weeks later (late monophasic fall) occurred in 7 children (3 %). Fall in haematocrit ≥5 units following treatment occurred in 57 children [23 %] between 14 and 28 days after treatment began. Baseline parasitaemia and proportion with > 100,000μL^-1 ^asexual forms were significantly higher in children with ≥5 units compared to <5 units fall in haematocrit 21 or 28 days after treatment began. Irrespective of pattern, declines in haematocrit deficit from <30 % were mono-exponential, with similar half-times for AA- and AL-treated children (1.32 d versus 1.14 d). Anaemia half-time correlated significantly positively with anaemia recovery time in the same patients (*r* = 0.55, *P* < 0.0001). Bland-Altman analysis of 9 or 10 multiples of anaemia half-time and anaemia recovery times showed narrow limit of agreement with insignificant biases (*P* = 0.19 or 0.63, respectively).

**Conclusions:**

In uncomplicated falciparum malaria, increases or falls in haematocrit are common following ACTs. Falls in haematocrit ≥ 5 units are common and may or may not result in early or late anaemia. In children who recovered from acute falciparum malaria-associated anaemia following ACTs, decline in haematocrit deficit is mono-exponential.

**Trials registration:**

Pan African Clinical Trial Registry PACTR201508001188143, 3 July 2015; PACTR201508001191898, 7 July 2015 http://www.pactr.org.

**Electronic supplementary material:**

The online version of this article (doi:10.1186/s12879-015-1219-y) contains supplementary material, which is available to authorized users.

## Background

Artemisinin-based combination treatments (ACTs) are the first line treatments of uncomplicated falciparum malaria in over 100 countries [1–4] and intravenous artesunate, considered superior to quinine [5, 6], is now the recommended treatment for severe falciparum malaria [7]. Recent case reports indicate intravenous artesunate may cause delayed haemolytic anaemia in immunologically naïve patients with severe falciparum malaria [8–11] and orally administered ACTs to a lesser extent may cause similar effect even in uncomplicated infections in children from endemic areas [11].

*Plasmodium falciparum* malaria-associated anaemia, is common in children [12, 13], occurs in 20 – 50 % of African children with falciparum infections [13–15], and is thought to be due to destruction of parasitized and non-parasitized red blood cells and bone marrow dyserythropoiesis of variable intensity and duration [16–18]. Two of the hallmarks of sensitive *Plasmodium falciparum* infections to ACTs, namely rapid clearance of asexual parasitaemia and recovery from the symptoms and signs of the infections [3, 7], are often followed by increases in haematocrit or haemoglobin in majority of children following recovery from acute infections [12, 13, 19, 20]. A recent study in Nigerian children with uncomplicated falciparum malaria-associated anaemia at presentation showed that time elapsing from commencement of ACTs until resolution of the associated anaemia (anaemia recovery time) was approximately 10 days and was unrelated to age [21].

Despite adoption of ACTs as first line treatments in many endemic countries, there is little information on the patterns of change in haematocrit in individual African children following ACTs of uncomplicated infections. Such information may not only assist with the community management of malaria-associated falls in haematocrit to <30 %, but also with understanding the extent of falls in haematocrit in the different endemic settings, the relationship between falls and time-course of treatment, and the characteristics of the children with different pattern of change in haematocrit following ACTs.

In the present study, the temporal patterns of haematocrit after artemisinin-based combination treatments of uncomplicated falciparum malaria were evaluated in a group of children resident in an endemic area of southwestern Nigeria. The main aims were to: (i) characterize the changes in haematocrit with time in the individual patients, (ii) evaluate the factors contributing to moderate fall (≥5 unit fall from baseline) in haematocrit following treatment, and (iii) characterize, kinetically, recovery from the fall in haematocrit below 30 % associated with the different patterns.

## Methods

### Study location

The study was a prospective study conducted between April 2008 and December 2011 in Ibadan, southwestern Nigeria- an endemic area. It was nested in a larger study of *Plasmodium falciparum* malaria-associated anaemia in children before, during and after artemisinin-based combination treatments (Pan African Clinical Trial Registry PACTR201508001188143 & PACTR201508001191898). The details of the larger study have been reported elsewhere [20, 22, 23]. The study protocols were approved by the Ministry of Health, Ibadan and the National Health Research Ethics Committee, Abuja, Nigeria.

### Patients

Briefly, patients were enrolled in the study if they were aged 6 months–15 years, had symptoms compatible with acute uncomplicated malaria with *Plasmodium falciparum* mono-infection ≥2000 μL^−1^ of blood, no history of antimalarial drug ingestion in the two weeks prior to enrolment, absence of severe malaria [24] and written informed consent given by parents or guardians.

Enrolled patients were randomized to receive artemether-lumefantrine or artesunate-amodiaquine (co-formulated). Artemether-lumefantrine (Coartem®, Novatis, Basel, Switzerland) was given according to body weight: patients weighing 5–14 kg received one tablet, those weighing 15–24 kg received two tablets, those weighing 25–34 kg received three tablets, and those weighing >34 kg received four tablets at presentation (0 hour), 8 hours later and at 24, 36, 48 and 60 hours after the first dose. Each tablet of artemether-lumefantrine contains 20 mg of artemether and 120 mg of lumefantrine. Artesunate-amodiaquine (Coarsucam®, Sanofi Aventis, France) was also given according to body weight: patients weighing ≥4.5 to <9 kg received one tablet, those weighing ≥9 to <18 kg received one tablet and those weighing ≥18 to <24 kg received one tablet of the following formulations: 25 mg/67.5 mg, 50 mg/135 mg, 100 mg/270 mg of fixed dose combination of artesunate/amodiaquine, respectively daily for 3 days. Children weighing 24–36 kg and >36 kg received 1.5 and 2 tablets, respectively of 100/270 mg of fixed dose combination of artesunate/amodiaquine daily for 3 days.

All drugs were given orally. All doses of artesunate-amodiaquine were given under direct observed therapy as were the doses of artemether-lumefantrine given at 0, 8, 24 and 48 hours. Doses of artemether-lumefantrine at 36 and 60 hours were given by parents/guardians of the children at home and enquiries were made by telephone calls at the expected times of administration to confirm that the doses were actually given. Artemether-lumefantrine was not given with fatty meals.

Thick and thin blood films prepared from finger prick were stained with Giemsa and examined by light microscopy under oil immersion objective lens 1000 x magnification by two assessors who did not know the drug regimen of the patients. A senior member of the study team reviewed the slides if there was any disagreement by the two microscopists. In addition, the slide of every fourth child enrolled in the study was reviewed by the senior member. Thick and thin blood films obtained from each child as soon as they came to the clinic on to blood slides were carefully labeled with the patients’ codes and air-dried before being stained. Follow-up with clinical and parasitological evaluation was done daily on days 1–3 and on days 7, 14, 21, 28, 35 and 42.

Parasitaemia, asexual or sexual, in thick films were estimated by counting asexual and sexual parasites relative to 500 leukocytes, or 500 asexual or sexual forms whichever occurred first. From this figure, the parasite density was calculated assuming a leukocyte count of 6000 μL^−1^ of blood. A slide was considered parasite negative if no asexual or sexual parasite was detected after examination of 200 microscope fields. The cure rates on days 21 and 28 were adjusted on the basis of the PCR (polymerase chain reaction) genotyping results of paired samples of patients with recurrent parasitaemia after day 7 of starting treatment as previously described [20].

Capillary blood collected before treatment and during follow-up was used to measure hematocrit using a microhaematocrit tube and microcentrifuge (Hawksley, Lancing, UK). Anaemia was defined as a haematocrit <30 % [12, 13]. Anaemia recovery time (in anaemic patients at presentation) was defined as time elapsing from drug administration to attainment of a hematocrit value ≥30 % [21, 25] in patients with anaemia at presentation. In patients who had early or late monophasic decline in haematocrit which resulted in anaemia, anaemia recovery time was defined as time from appearance of, to recovery from, anaemia. Children were also considered to have late fall in haematocrit attributable to treatment if they had no concomitant illness during the period of the fall and were parasite negative by both microscopy and PCR.

### Evaluation of temporal changes in haematocrit following treatment

**Fig. 1:**
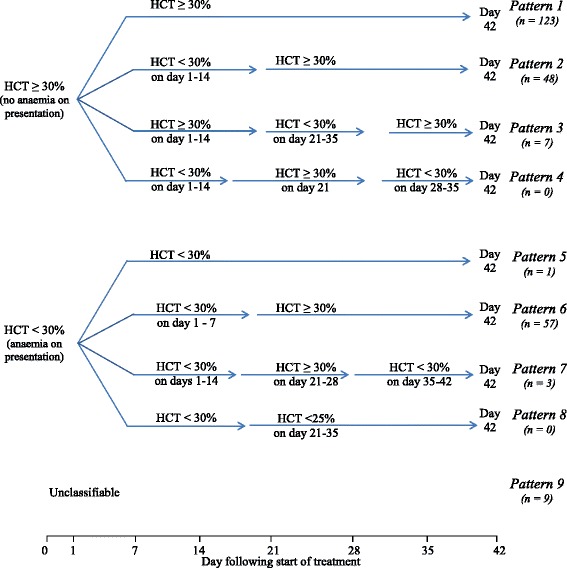
Classification of temporal patterns of haematocrit after artemisinin-based combination treatments. HCT, haematocrit

Haematocrit <30 % and ≥30 % were the reference points in all classified patterns. Temporal changes in haematocrit were classified into the following patterns (Fig. 1).

1. Haematocrit ≥30 % before and following treatment.
2. Haematocrit ≥30 % before treatment, declining to haematocrit <30 % within 2 weeks of treatment, and thereafter increasing to ≥30 % by 6 weeks after treatment (early monophasic fall).
3. Haematocrit ≥30 % before treatment followed by a decline to haematocrit <30 %, 3–5 weeks following treatment and then increasing to ≥30 % by 6 weeks after treatment (late monophasic fall).
4. Haematocrit ≥30 % before treatment, 2 consecutive haematocrit <30 % within 14 days of treatment, followed by a rise to haematocrit ≥30 % between 14 and 21 days and a fall to <30 % between day 28–35 (biphasic fall in haematocrit).
5. Haematocrit <30 % before and following treatment (persistent anaemia).
6. Haematocrit <30 % before treatment followed by an increase to ≥30 % after treatment (malaria-associated anaemia at presentation and recovery from anaemia).
7. Haematocrit < 30 % at presentation and till day 14, followed by a rise to ≥30 % on days 21–28 and a fall to <30 % on days 35–42.
8. Haematocrit <30 % before treatment, followed by further falls to ≤25 %, 3–5 weeks following treatment.
9. Unclassifiable.

### Kinetics of the disposition of deficit in haematocrit from 30 %

The kinetics of the disposition of deficit in haematocrit from 30 %, that is, of anaemia, was as previously described [20, 26]. Briefly, in all anaemic patients at enrolment, or when anaemia occurs following treatment, haematocrit values below 30 % (the lower threshold of normal) and at follow-up were subtracted from 30 % at each time of measurement until haematocrit rose to ≥30 %, and the resulting values plotted against time. The final haematocrit when anaemia resolved was therefore zero in all patients. However, the final haematocrit at the time of resolution was assumed to be 0.01 %. The areas under the curve (AUC) of deficit in haematocrit (from 30 %) *versus* time were obtained, by the trapezoidal rule using the computer program *Turbo Ken* (designed by Clinical Pharmacology Group, University of Southampton, United Kingdom) as previously described (20, 26). AUC was also obtained manually by calculating the average haematocrit values between two consecutive time measurements and multiplying it by the time interval between the measurements, and summing up all the values, in a manner similar to that for the numerical estimation of area under a drug concentration-time curve [27]. Both measurements by digital computer and manual methods gave the same values. The unit of quantification would be %.d, if hematocrit values were used or g/L.d if hemoglobin values were used. Hematocrit values may be converted to hemoglobin values by dividing by 3 [28]. The apparent terminal elimination rate constant (λ) was obtained by least-square regression analysis of the post-peak log-linear part of the plot of deficit in hematocrit (from 30 %) *versus* time, and the apparent terminal half-time of anaemia (t_1/2(anaemia)_) was calculated from ln/(λ).

### Statistical analysis

Data were analyzed using version 6 of Epi-Info software [29] and the statistical program SPSS for Windows version 20.0. [30]. Variables considered in the analysis were related to the densities of *P. falciparum* asexual and sexual forms. Proportions were compared by calculating χ^2^ using Yates’ correction, Fisher’s exact or Mantel Haenszel tests. Normally distributed, continuous data were compared by Student’s t test and analysis of variance (ANOVA). Data not conforming to a normal distribution were compared by the Mann-Whitney U tests and the Kruskal Wallis tests. The relationship between two continuous variables was assessed by Spearman’s rank correlation coefficient. Agreement between pharmacodynamic and pharmacokinetic methods of evaluating recovery from anaemia was assessed by Bland-Altman analysis [31]. *P*-values of <0.05 were taken to indicate significant differences. Data were double entered serially using patients’ codes and were only analyzed at the end of the study.

## Results

### Patients’ characteristics and therapeutic responses

Between April 2008 and December 2011, 1180 patients were enrolled in prospective studies of the efficacy of artemisinin-based combination treatments. All patients, at enrolment and during follow-up, had haematocrit measured. However, two hundred and forty eight patients who had haematocrit measured during the entire period of follow-up were analysed. Of these, 177 and 71 were treated with artesunate-amodiaquine and artemether-lumefantrine, respectively. The clinical characteristics of the patients at enrolment are summarized in Table 1. Forty four per cent of the patients were aged less than 5 years; hyperparasitaemia (>250,000 μL^−1^) was found in 8.9 % of the patients. In general, PCR-corrected cure rates were similar for both artesunate-amodiaquine and artemether-lumefantrine (98.3 % [95 % CI 96.1-99.3 %] *versus* 96.9 % [95 % CI 93.9-98.2 %] for artesunate-amodiaquine and artemether-lumefantrine, respectively).

**Table 1:** Characteristics of the 248 children enrolled in the study

	Value
No. of children	248
Female (%)	117 (47)
aged < 5 years	110
Febrile (> 37.4 °C)	119
Parasitaemia	
(> 100,000 μL^−1^)	71
(> 250,000 μL^−1^)	22
Anaemia	
(< 30 %)	67
(< 15 %)	0
Enlarged liver	50
Enlarged spleen	26
Mean value (range) for	
Age (year)	6.3 (0.7–14)
Weight (kg)	18.0 (7–40)
Duration of illness (day)	2.7 (1–7)
Temperature (°C)	38.4 (35–40.9)
Haematocrit (%)	31.9 (18–42)
GMPD (μL^−1^ of blood)	57,504 (2,000–1,080,200)
Liver enlargement (cm) [*n* = 50]	2.9 (0.1–8)
Spleen enlargement (cm) [*n* = 26]	1.5 (0.3–6)

GMPD = geometric mean parasite density

### Temporal changes in haematocrit following start of treatment

Tables 2 and 3 show the distribution of patients and their characteristics in the different temporal patterns. Children who were anaemic at presentation (Pattern 6), were younger, weighed significantly less, had significantly longer duration of illness and a significantly higher proportion of children who were hyperpyrexia compared to children without anaemia (Patterns 1 and 2) (Table 2). However, bodyweight was similar in children in patterns 2 and 6 (by *post-hoc* comparison). Parasitaemia was similar in all three temporal patterns. The features of the other temporal patterns (Patterns 3, 5 and 7) are summarized in Table 3. The features appear similar due to the small number of patients in each pattern. No patient could be classified as pattern 4 or 8. Nine patients could not be classified into any of the patterns described above (unclassifiable).

**Table 2:** Clinical, parasitological and other characteristics of patients in different major temporal patterns

	^a^Pattern 1	^b^Pattern 2	^c^Pattern 6	ALL	^e^ *P* value
Number (%)	123 (49.6)	48 (19.4)	57 (23)	228 (92)	
Gender					
M/F	66/57	21/27	33/24	120/108	0.33
Age (year)					
Mean (sd)	7.3 (3.6)	5.6 (2.7)	5.3 (2.7)	6.5 (3.3)	< 0.0001
Range	0.7–14	2–10	1.2–12.5	0.7–14	
Duration of illness (days)					
Mean (sd)	2.5 (1.1)	2.8 (1)	3.2 (1.3)	2.7 (1.2)	< 0.0001
Range	1–7	1–5	1–7	1–7	
Weight (kg)					
Mean (sd)	20.2 (7.5)	16.7 (5.3)	16.0 (4.3)	18.4 (6.7)	0.009
Range	7–40	9–31	7–26	7–40	
Haematocrit (%)					
Mean (sd)	34.7 (2.7)	32.4 (2.8)	26.4 (2.6)	32.3 (4.4)	< 0.0001
Range	30–42	30–38	18–29	18–42	
Temperature (°C)					
Mean (sd)	38.4 (1.1)	38.6 (1)	38.4 (1.2)	38.4 (1.1)	0.60
Range	35–40.9	35–40.1	35.9–40.5	35–40.9	
No. with temp.					
i. >37.4 °C (%)	97 (79)	42 (88)	43 (75)	182 (80)	0.28
ii. >40.0 °C (%)	8 (7)	1 (2)	14 (25)	23 (10)	< 0.0001
Parasitaemia (μL^−1^)					
Geometric Mean	52,037	59,361	64,514	56,468	0.37
Range	2,000-459,000	8,000-1,080,200	2,094-346,153	2,000-1,080,200	
No. with ≥100,000 μL^−1^(%)	34 (27.6)	11 (22.9)	18 (31.6)	63 (27.6)	^d^0.61

^a^Haematocrit ≥ 30 % before and following treatment

^b^Haematocrit ≥ 30 % before treatment, two consecutive haematocrit < 30 % within 14 days of treatment, followed by a rise to ≥ 30 % [Early monophasic fall]

^c^Haematocrit < 30 % before treatment, followed by a rise to ≥ 30 % after treatment [malaria-associated anaemia at presentation and recovery from anaemia]

^d^Chi-square test

^e^ *P* values quoted are for comparison across all three groups

**Table 3:** Clinical, parasitological and other characteristics of patients in different minor temporal patterns^d^

	^a^Pattern 3	^b^Pattern 5	^c^Pattern 7
Number (%)	7 (2.8)	1 (0.4)	3 (1.2)
Gender			
M/F	4/3	0/1	2/1
Age (year)			
Mean (sd)	5.6 (2)	2	2.7 (0.3)
Range	2–8	2	2.5–4
Duration of illness (days)			
Mean (sd)	2.5 (0.8)	3	2.3 (0.6)
Range	1–3	3	2–3
Weight (kg)			
Mean (sd)	17 (3.1)	10	11.7 (2.5)
Range	13–20	10	9–14
Haematocrit (%)			
Mean (sd)	34.1 (3.6)	22	27.3 (2.1)
Range	30–40	22	25–29
Temperature (°C)			
Mean (sd)	38.9 (1.3)	37.1	38.8
Range	36.7–40.6	37.1	38.8
No. with temp.			
>37.4 °C (%)	6 (86)	0 (0)	3 (100)
>40.0 °C (%)	2 (29)	0 (0)	0 (0)
Parasitaemia (μL^−1^)			
Geometric mean	67,985	288,461	65,333
Range	14,792-197,308	288,461	21,220-467,681
No. with >100,000 μL^−1^ (%)	3 (43)	1 (100)	1 (33)

^a^Haematocrit ≥ 30 % before treatment followed by haematocrit < 30 % 3 – 5 weeks following treatment, and a rise to ≥ 30 % by 6 weeks after treatment

^b^Haematocrit < 30 % before and following treatment

^c^Haematocrit < 30 % before treatment, followed by > 30 % by days 21–28, and thereafter a fall to <30 %, 5 – 6 weeks following treatment

^d^Statistical comparison not carried out because of very few number of patients in the three patterns

**Fig. 2:**
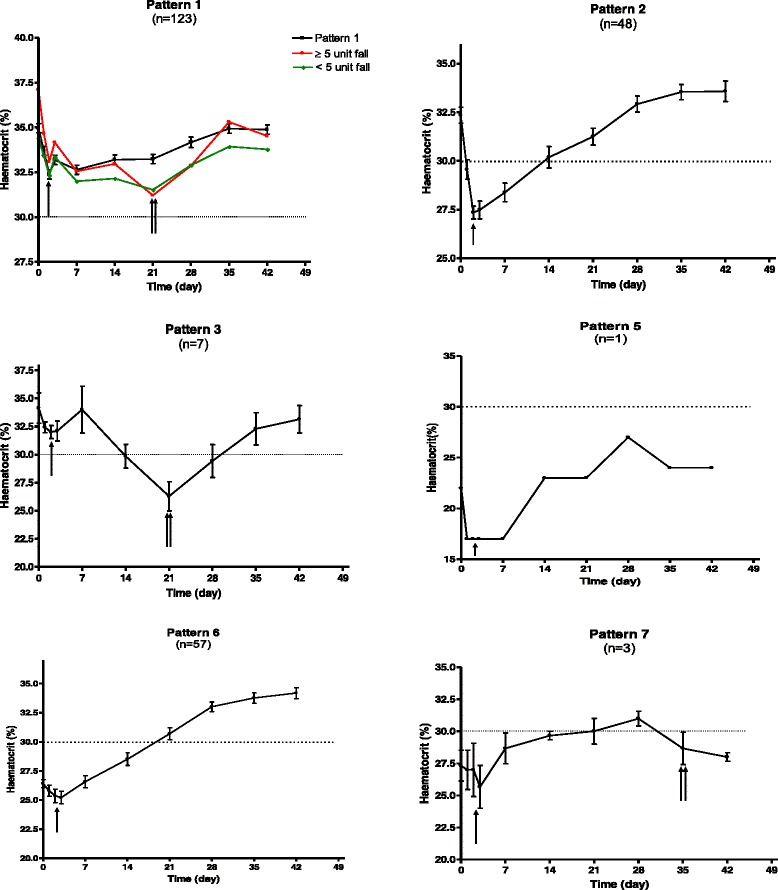
Temporal patterns of haematocrit after artemisinin-based combination treatments. Single arrow indicates drug attributable fall, and double arrows indicate late fall in haematocrit even in patients with normal haematocrit during entire period of follow up (see Pattern 1)

In all temporal patterns described (Fig. 2), a prominent feature is a fall in haematocrit within the first three days of drug administration–the drug-attributable fall in haematocrit (DAFH [25]). A late fall in haematocrit which began after day 14 and persisted beyond 28 days after treatment began was seen in Patterns 1, 3 & 7 (Fig. 2). The detailed clinical and parasitological characteristics and outcome of treatment of patients with a late fall in haematocrit resulting in anaemia between days 21 and 35 are shown in Table 4. At enrolment, 4 of the 7 patients had parasitaemia >100,000 μL^−1^, parasitemia cleared within 1 day in 6 of 7 children, 3 of 5 children treated with artesunate-amodiaquine had total dose of artesunate >11 mg/kg, and none of the children required blood transfusion. Also, none of the patients was older than 9 years. On the whole, virtually all of these children were anaemic for a period of approximately 14 days (Additional file 1: Figure S1). The mean duration of anaemia was 16.0 days (95 % CI 12.8–19.2; range 14–21 days).

**Table 4:** Features of patients with late fall in haematocrit below 30 % (Pattern 3)

Patient (gender, age)	Year of enrolment	Parasitaemia (μL^−1^)	Enrolment HCT (%)	Antimalarial treatment	PCT (day)	Nadir HCT(%)^a^ (day)	HCT on day 42 (%)	Outcome AnRT (day)	Total of mg/kg dose of AA		Total of mg/kg dose of AL	
									Artesunate	amodiaquine	artemether	lumefantrine
13 (F, 4.9y)	2010	357,397	40	AA	1	27 (21)	30	14	11.5	31.2	NA	NA
20 (M, 4.9y)	2010	197,308	30	AL	1	20 (21)	40	14	NA	NA	9.2	55.4
28 (F, 8y)	2010	157,200	37	AA	1	27 (14)	33	14	17.5	30.0	NA	NA
45 (M, 5.4y)	2009	34,000	30	AL	2	28 (21)	32	21	NA	NA	15	90
93 (F, 7.8y)	2009	35,593	34	AA	1	23 (21)	31	21	7.5	23.0	NA	NA
167 (M, 6y)	2009	14,792	34	AA	1	29 (21)	33	14	16.7	51.0	NA	NA
200 (M, 2y)	2009	181,000	34	AA	1	28 (14)	33	14	10.5	42.6	NA	NA

^a^Percentage fall in haemaocrit compared to pre-treatment values in descending order of cases are: 40 %, 33 %, 33 %, 32 %,27 %, 18 %, and 15 %, respectively

*NA* = Not Applicable; *AA* = artesunate-amodiaquine; *AL* = artemether-lumefantrine; *HCT* = Haematocrit, *PCT* = Parasite clearance time

*AnRT* = anaemia recovery time

### Comparison of children with fall in haematocrit ≥5 units and <5 units following treatment

Because of very few patients with late fall in haematocrit (n = 7), the features of children with ≥5 units and <5 units fall in haematocrit from baseline following artemisinin-based combination treatments were evaluated. Based on the number of patients evaluated per visit day, on day 7, 16 of 23 children and 62 of 87 children had ≥5 and <5 units fall in haematocrit, respectively, following treatment. The difference between these proportions was not significant (χ^2^ = 0.02; *P* = 0.92). After day 7, overall, 57 of 122 children and 55 of 213 children with ≥5 and <5 units fall in haematocrit, respectively between days 14 and 28 were anaemic (Table 5). The difference between these proportions was significant (χ^2^ = 14.3, *P* = 0.0001). However, the proportion of children who were anaemic on day 14 in the 2 groups, respectively were similar (29 of 52 *v* 32 of 65, χ^2^ = 0.3, *P* = 0.60). On day 21, 20 of 48 children and 18 of 75 children in the 2 groups, respectively were anaemic (χ^2^ = 4.2, *P* = 0.04 by Mantel Haenszel test). On day 28, 8 of 22 and 5 of 73 children (Table 5) in the 2 groups, respectively were anaemic (χ^2^ = 10.1, *P* = 0.0001).

**Table 5:** Fall in haematocrit according to time of follow-up in children treated with artesunate-amodiaquine or artemether-lumefantrine

	Days after start of treatment									
Extent of fall	14 Anaemia		21 Anaemia		28 Anaemia		35 Anaemia		42 Anaemia	
	Yes	No	Yes	No	Yes	No	Yes	No	Yes	No
≥ 5 % fall in haematocrit	29^a^	23^a^	20	28	8	14	0	11	1	15
< 5 % fall in haematocrit	32	33	18	57	5	68	5	62	3	61
Total	61	56	38	85	13	82	13	82	4	76

^a^Number of children evaluated per visit day

Table 6 shows the clinical and parasitological parameters of the 89 children who had a fall of ≥5 or <5 unit in haematocrit from baseline on days 21 or 28, or on both days. Children with ≥5 units fall in haematocrit had significantly higher baseline haematocrit, parasitemia and proportion of children with asexual forms >100,000 μL^−1^. However, median doses of artesunate were similar in the two groups. Similarly, the median doses of artemether, lumefantrine or amodiaquine were similar in the two groups. In a univariate analysis (see Additional file 2: Table T1), a parasitaemia >100,000 μL^−1^ at enrollment was significantly associated with ≥5 units fall in haematocrit from baseline on days 21 or 28 or on both days.

**Table 6:** Clinical, parasitological and other characteristics of patients with falls in haematocrit ≥5 and <5 units on day 21, 28 or both following treatment with artesunate-amodiaquine or artemether-lumefantrine

	≥5 units fall in haematocrit^a^	<5 units fall in haematocrit	*P* value
Number	51	38	
M/F	27/24	22/16	0.72
Age (year)			
Mean (sd)	6.6 (3.1)	6.8 (3.3)	0.73
Range	0.7 – 13.0	2.5–14	
No. < 5 years	19	18	0.46
Duration of illness (days)			
Mean (sd)	2.6 (1)	2.6 (0.9)	0.96
Range	1–5	1–5	
Weight (kg)			
Mean (sd)	18.8 (6.7)	19.1 (6.2)	0.82
Range	7–37	11–36	
Haematocrit (%)			
Mean (sd)	35.9 (3.3)	33.1 (3)	< 0.0001
Range	29–42	24–38	
Temperature (°C)			
Mean (sd)	38.2 (1.1)	38.5 (1.3)	0.26
Range	35.0–40.4	36.0–40.9	
No. with temperature			
>37.4 °C	39	31	0.68
>40.0 °C	3	5	0.25
Parasitaemia(/μL)			
Geometric mean	72,208	44,751	0.026
Range	3,529–501,946	3,749–281,538	
No. with parasitaemia			
> 100,000/μL	22	6	0.003
> 250,000/μl	5	2	0.42
PCT (day) (sd)	1.1 (0.2)	1.2 (0.4)	0.14
No. with parasitaemia on day 1	3	6	0.15
Median dose (mg/kg)			
Artesunate (range)	12.5 (5–40) [*n* = 43]	11.1 (4.7–18) [*n* = 24]	0.12
Amodiaquine (range)	33.5 (15.3–76.5) [*n* = 43]	30.9 (14.3–55.1) [*n* = 24]	0.18
Artemether (range)	10.7 (9.2–16) [*n* = 8]	12.4 (7.1–16) [*n* = 11]	0.78
Lumefantrine (range)	64.3 (55.4–96) [*n* = 8]	68.6 (42.4–90) [*n* = 11]	0.78

^a^Six of the seven children with late fall in haematocrit belong to this group (see also Table 4)

### Kinetics of the disposition of the deficit in haematocrit from 30 %

#### (a). Comparison of the kinetics of the disposition of deficit in haematocrit in children with early (Pattern 2) and late (Pattern 3) monophasic falls in haematocrit

Forty-eight (31 treated with artesunate-amodiaquine and 17 treated with artemether-lumefantrine) and 7 (5 treated with artesunate-amodiaquine and 2 treated with artemether-lumefantrine) children, respectively, had early and late monophasic falls in haematocrit. The children from the two groups, respectively had similar clinical and parasitological parameters at presentation (Age 5.6 ± 2.7 years [range 2–14] *versus* 5.6 ± 2.0 years [range 2–8], P = 0.95; duration of illness 2.8 ± 1.0 d [1–5] *versus* 2.5 ± 0.8 d [1–3], *P* = 0.45; body temperature 38.6 ± 1.0 °C [range 35.0–40.1] *versus* 38.9 ± 1.3 °C [range 36.7–40.6], *P* = 0.38; haematocrit 32.4 ± 2.8 % [range 30–40] *versus* 34.1 ± 3.6 % [range 30–40], *P* = 0.13, and geometric mean asexual parasite density 59,361 μL^−1^ blood [range 8,000–1,080,200] *versus* 67,895 μL^−1^ blood [range 14,792–197,308], *P* = 0.74). The median doses of the drugs were also similar in the two groups (data not shown). Time to recover from anaemia was significantly shorter in children with early than in those with late monophasic falls in haematocrit (median 11 d [range 2–27; n = 48] *versus* 14 d [range 14–21; n = 7], *P* = 0.003). The mean AUC of deficit in haematocrit *versus* time was significantly lower in children with early monophasic fall than in those with late monophasic fall (mean 14.7 %.d [range 3.7–41.5] *versus* 54.1 %.d [range 20.2–116.4], *P* = 0.002). Declines of deficit in haematocrit were monoexponential in both patterns (see Additional file 3: Figure S2). The half-time of decline was significantly shorter in children with early monophasic fall than in those with late monophasic fall in haematocrit (mean 0.9 ± 0.7 d [range 0.2–2.1] *versus* 1.6 ± 0.4 d [range 1.1–2.1], *P* = 0.003).

#### (b). Anaemia at presentation followed by recovery (Pattern 6)

**Fig. 3:**
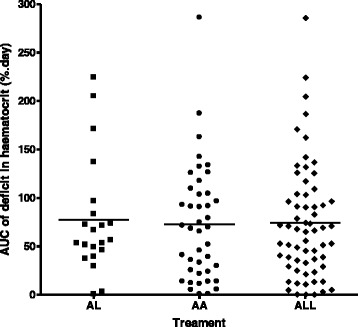
Distribution of individual area under curve (AUC) of deficit in haematocrit from 30 % in children with anaemia at presentation (Pattern 6), after treatment of falciparum infections with artemether-lumefantrine (AL) or artesunate-amodiaquine (AA). ALL; all children. Horizontal bars indicate mean AUC

The demographic and other characteristics of the 57 children (n = 39 for artesunate-amodiaquine and n = 18 for artemether-lumefantrine) have been summarized in Table 2. Overall, in all children, mean AUC of deficit in haematocrit *versus* time was 74.2 %.day (95 % CI 59.3–89.1, range 0.7–286.2) and it was similar in anaemic children treated with artesunate-amodiaquine and artemether-lumefantrine (mean 72.7 %.day [95 % CI 54.1–91.2, range 0.7–286.2] *versus* mean 77.3 %.day [95 % CI 50.1–104.6, range 0.7–224.6], *P* = 0.77) (Fig. 3). Overall, in all children, there was mono-exponential decline of the deficit in haematocrit (see Additional file 4: Figure S3) with an estimated half-time (t_½_) of 1.3 d (95 % CI 1.2 – 1.5, range 0.2–2.5). Estimated mean t_½_ were similar in children treated with artesunate-amodiaquine and artemether-lumefantrine (mean 1.3 d [95 % CI 1.1–1.5, range 0.2–2.5] *versus* mean 1.3 d [95 % CI 1.1–1.6, range 0.2–2.1], *P* = 0.93).

#### (c) Relationship between half time of decline in haematocrit deficit and anaemia recovery time

**Fig. 4:**
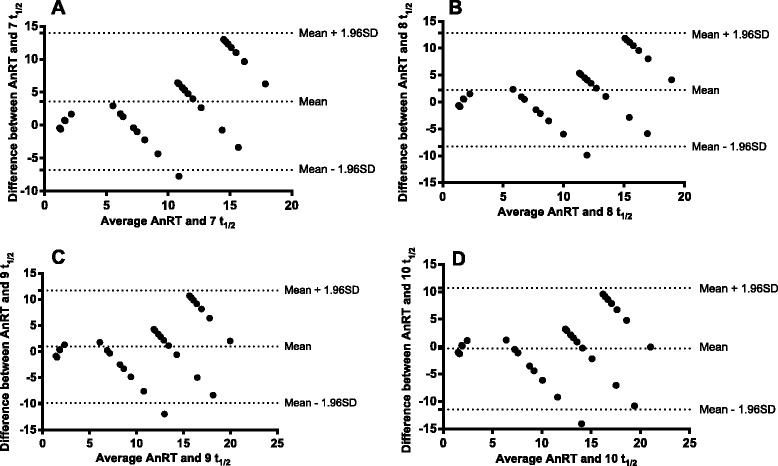
Bland-Altman plots of anaemia recovery times and multiples [7 **a** 8 (**b**), 9 (**c**) and 10 (**d**)] of anaemia half-times. Biases were 3.62, 2.29, 0.96 and −0.36 for plots A, B, C and D; *P* < 0.0001, *P* = 0.002, 0.19 and 0.63, respectively. The mean values ± 1.96 standard deviation (SD) of the differences are shown

The relationship between the half-time of decline in haematocrit deficit from 30 % and anaemia recovery time in the same patients with anaemia at presentation was evaluated in 57 children. The mean half-time of decline in haematocrit deficit from 30 % was 1.3 days (95 % CI 1.2–1.5, range 0.2–2.5). The median anaemia recovery time was 14 days (range 1–21). There was a significantly positive correlation between half-time of decline in haematocrit deficit from 30 % and anaemia recovery time (*r* = 0.55, *P* < 0.0001, see Additional file 5: Figure S4). The ratio of anaemia recovery time to half time of decline in haematocrit in individual patient was 10.0 (95 % CI 9.0–11.1). Bland-Altman plots of the anaemia recovery times and multiples of anaemia half-times are shown in Fig. 4. The limits of agreement between anaemia recovery times and 9 or 10 multiples of anaemia half-times were narrow. At 9 and 10 anaemia half-time, the limits of agreement were −9.8–12 days and −11–11 days, respectively. The bias at multiples of 9 or 10 anaemia half-times was statistically insignificant (*P* = 0.19 and 0.63, respectively). However, there was a statistically significant bias at multiples of 7 or 8 anaemia half-times (*P* < 0.0001 and *P* = 0.002, respectively).

## Discussion

In this study, in keeping with previous reports, the commonest changes in haematocrit after successful artemisinin-based combination treatments of uncomplicated falciparum infections in children are increases in haematocrit in non-anaemic (Pattern 1) and anaemic (Pattern 6) children at presentation [12, 13, 19, 20, 23, 25, 32]. When combined with children who had no anaemia – early anaemia – no anaemia pattern (Pattern 2, early monophasic fall in haematocrit), over 90 % of the children could be classed into one of these three patterns. Compared to children with normal haematocrit at presentation and following treatment (Pattern 1) and those with early monophasic fall in haematocrit (Pattern 2), children with anaemia – no anaemia pattern (Pattern 6, acute falciparum malaria-associated anaemia at presentation and recovery from anaemia) were significantly younger and had significantly longer duration of illness in keeping with previous findings [12, 13, 15]. However, baseline parasitaemia was similar in all the three patterns, − a finding in anaemia – no anaemia pattern that is at variance with some reports showing high parasitaemia is a risk factor for anaemia at presentation in children with acute falciparum malaria [12, 13, 15].

Of theoretical interest, for which no child was classified is: no anaemia at presentation, followed by anaemia, recovery from anaemia, and a late fall in haematocrit to anaemia level (Pattern 4). This pattern appears to have combined features of Pattern 2 and Pattern 3 and may be considered a variant of both Patterns 2 and 3. It would appear that the 3 patients included under pattern 7 appeared to have ‘undulating pattern’. We do not have an explanation for this pattern.

It is noteworthy that a late fall in haematocrit to a level below normal occurred in 3 % of the children (Pattern 3, Table 4). Over half of the children with late fall in haematocrit had high parasitaemia (>100,000/μL), virtually all their parasitaemias cleared by 1 day after treatment started and 3 of 5 children treated with artesunate-amodiaquine had >11 mg/kg of artesunate. With the exception of overt clinical and other laboratory manifestations of haemolysis, the features of these children superficially resemble those of immunologically naïve patients with delayed haemolytic anaemia following intravenous artesunate treatment of severe falciparum malaria [8–11]. In some of the children with late fall in haematocrit who were treated with artemether-lumefantrine, the dose of artemether was not particularly high. It is likely that the children who received higher dose of artesunate were on the lower segment of the dose range since co-formulated preparations were used. Thus, in uncomplicated falciparum malaria when compared with severe malaria, the late fall in haematocrit to anaemia level may be associated with subtle clinical or laboratory features of haemolysis.

All of the factors associated with late monophasic fall in haematocrit after artemisinin-based combination treatments of uncomplicated falciparum malaria are not known. In order to understand some of these factors, a comparison of falls in haematocrit from baseline ≥5 units or <5 units after treatment was assessed by comparing the features of patients in the 2 groups. Approximately half of the patients with falls in haematocrit ≥5 units had their falls 21–28 days after treatment began (see Table 5). In these patients, the ≥5 units fall was associated with significantly higher baseline parasitaemia, a significantly higher proportion of patients with a parasitemia in excess of 100,000 asexual forms/μL, but not significantly higher doses of all the combination drugs compared with children who had <5 units fall in haematocrit (from baseline) following treatment. These findings superficially resemble those of the reported cases of late haemolytic anaemia following intravenous artesunate treatment of severe falciparum malaria [9, 10]. Since 40 % of the children with falls in haematocrit ≥5 units between 21 and 28 days after treatment began developed late monophasic anaemia as compared to 16 % of those with <5 units fall in haematocrit during the same period, it follows that late fall in haematocrit from baseline ≥5 units may or may not result in anaemia. This may explain the relatively low prevalence of late monophasic anaemia following artemisinin-based combination treatments in general. In this context, studies on the risk factors for late monophasic anaemia after artemisinin-based combination treatments of uncomplicated falciparum malaria in children are urgently needed in this endemic area.

In the area of study, the relatively common occurrence of the haemoglobinopathies, glucose-6-phosphate dehydrogenase deficiency, and intestinal helminth infections [33–36] may contribute to the background low haematocrit or even to the early monophasic decline in haematocrit following treatment but the extent may be difficult to quantify. However, they are less likely to contribute much to the late monophasic fall in haematocrit. The anaemia associated with falciparum malaria is multifactorial and is thought to be due to greater destruction of unparasitized red blood cells compared to parasitized red blood cells, and bone marrow dyserythropoiesis of variable intensity and duration [16–18, 37–39]. It is likely that the late monophasic fall in haematocrit (Pattern 3) following artemisinin-based combination treatments, is due to the late destruction of red blood cells from which parasites have been removed in the spleen by pitting-the parasite-negative-red cell surface antigen positive red cells [40]. These red blood cells are found in the circulation in large numbers within 24 hours of initiating treatment with artesunate, but they have shortened survival time in severe malaria compared with unparasitized red blood cells [41] and are therefore expected to be destroyed 3–5 weeks after treatment began [10, 11]. Thus, in patients with high parasitaemia, the fall in haematocrit in the first few days following artemisinin-based combination treatments is less than expected were all the parasitized red blood cells to be destroyed [42, 43]. It is also remotely possible that, in addition to these once infected red blood cells, apparently non-infected red blood cells may also be destroyed.

Recent case definition of post-artesunate delayed haemolytic (PADH) syndrome [44, 45] consists of 10 % fall in haemoglobin associated with haptoglobin <0.1 g/L and either an increase in LDH to >390 IL/L or a 10 % rise >7 days after start of treatment with artesunate in naïve patients with severe malaria. This syndrome has been reported in 7 % of African children with severe malaria treated with parenteral artesunate [46]. Although the seven children with delayed fall in haematocrit in our series did not meet this strict case definition, it is likely that the syndrome of delayed fall in haematocrit, not associated with severe malaria may occur in children with uncomplicated falciparum malaria following artemisinin-based combination treatments. In this context, the recent introduction and use of dihydroartemisinin-piperaquine (DHP) for the treatment of uncomplicated falciparum malaria in endemic areas of Africa would require close monitoring for the syndrome of delayed fall in haematocrit or heamoglobin as reported in 3 % of children following artemisinin-based combination treatments in this endemic area. This would be necessary since artesunate and artemether are metabolized to dihydroartemisinin. In addition, monitoring may help define the risk factors for artemisinin-related late falls in haematocrit following treatment of uncomplicated falciparum malaria in children.

Declines in haematocrit deficit from 30 % (that is, recovery from anaemia) were mono-exponential irrespective of whether the deficit was early or late, suggesting that using a non-compartment model, recovery from apparently uncomplicated falciparum malaria-associated anaemia is a first order process. In this and other contexts, early fall in haematocrit to <30 % differs from late fall to <30 % by its shorter half-time of anaemia. These two patterns also differ in their anaemia recovery times and AUC of deficit in haematocrit versus time. These differences suggest that, on the whole, the late monophasic anaemia may be more ‘intense’ than the early monophasic anaemia. A further application of pharmacokinetic principles should permit treatment of the increases in haematocrit associated with artemisinin-based combination treatments of uncomplicated falciparum malaria.

In this study, a pharmacodynamic-pharmacokinetic approach was used to quantify the time to resolve uncomplicated malaria-associated anaemia following artemisinin-based combination treatments. The approach showed the following: (i). Anaemia half-time correlated significantly with anaemia recovery time in the same patient. (ii). Greater than 8 multiples of anaemia half-times showed an insignificant bias when a Bland-Altman analysis was used. Thus, confirming that >8 multiples of anaemia half-times can be used interchangeably with anaemia recovery time for the evaluation of recovery from uncomplicated malaria-associated anaemia following artemisinin-based combination treatments. Therefore, the agreement between 9 or 10 multiples of anaemia half-times and anaemia recovery time was not unexpected since it takes 9 or 10 half-times for 99.6 or 99.9 % completion of an elimination process in a simple one compartment pharmacokinetic model. The ratio of anaemia recovery time to anaemia half-time of approximately 10 in the patients also indirectly support the model.

There are limitations of the present study. First, although the clinical and parasitolgical features of children with fall in haematocrit to anaemia level on days 21–42 after commencement of treatment were characterised, the nature of the anaemia was not fully characterized (i.e. whether it was haemolytic or not in nature). Second, in the children with late fall in haematocrit to anaemia level, quantification of once-infected and infected red blood cells and the disposition of these red cells during the course of follow-up was not evaluated. Third, The precise contribution of the background causes of anaemia in children from the endemic area (such as the haemoglobinopathies, malnutrition and intestinal helminth infections) to the time-course of haematocrit was not assessed.

## Conclusions

Increases in haematocrit are common following artemisinin-based combination treatments of uncomplicated falciparum malaria, but falls in haematocrit from baseline ≥5 units are also relatively common and may or may not result in early or late anaemia. In children who recovered from acute falciparum malaria-associated anaemia following artemisinin-based combination treatments, declines in haematocrit deficit (from 30 %, the lower threshold of normal) are mono-exponential.

## Additional files

Additional file 1: Figure S1. Haematocrit values of patients with late fall after artemisinin-based combination treatments of acute uncomplicated falciparum malaria. (DOCX 40 kb) Additional file 2: Table T1. Risk factors for ≥5 units fall in haematocrit from baseline in children with uncomplicated falciparum malaria treated with artesunate-amodiaquine or artemether-lumefantrine. (DOCX 12 kb) Additional file 3: Figure S2. Semilog plots of deficit in haematocrit from 30 % versus time in children with early monophasic fall (Pattern 2) [A] or late monophasic fall in haematocrit (Pattern 3) [B] following artemisinin-based combination treatments of uncomplicated falciparum infections. (DOCX 24 kb) Additional file 4: Figure S3. Semilog plots of deficit in haematocrit from 30 % versus time in children with haematocrit <30 % at presentation (Pattern 6). (DOCX 16 kb) Additional file 5: Figure S4. Scatter plots of half-time of the decline in haematocrit deficit from 30 % and anaemia recovery time children who were anaemic at presentation (*r* = 0.55, *P* < 0.0001). (DOCX 36 kb)

## Abbreviations

%: Percent
μL: Microliter
AA: Artesunate-amodiaquine
ACTs: Artemisinin-based combination treatments
AL: Artemether-lumefantrine
AnRT: Anaemia recovery time
AUC: Area under curve
D: day
DAFH: Drug attributable fall in haematocrit
DHP: Dihydroartemisinin-piperaquine
FCT: Fever clearance time
G: Gram
GMPD: Geometric mean parasite density
HCT: Haematocrit
Kg: Kilogram
L: Litre
M/F: Male/female
Mg: Milligram
°C: Degree Celsius
PCR: Polymerase chain reaction
PCT: Parasite clearance time
sd: Standard deviation
t_½_: Half-time

## References

[1] World Health Organization (2001). The use of Antimalarial drugs. Report of a WHO informal consultation. Document WHO/CDS/RBM/2001.33.

[2] World Health Organization (2001). Antimalarial drugs combination therapy. Report of a WHO technical consultation. Document WHO/CDS/RBM/2001.35.

[3] White NJ (2008). Qinghaosu (artemisinin): the price of success. Science.

[4] Bosman A, Mendis KN (2007). A major transition in malaria treatment: the adoption and deployment of artemisinin-based combination therapies. Am J Trop Med Hyg.

[5] Dondorp A, Nosten F, Stepniewska K, Day N, White N (2005). Artemisinin versus quinine for treatment of severe falciparum malaria: a randomised trial. Lancet.

[6] Dondorp AM, Fanello CI, Hendriksen IC, Gomes E, Seni A, Chhaganlal KD (2010). Artesunate versus quinine in the treatment of severe malaria in African children (AQUAMAT): an open-label, randomised trial. Lancet.

[7] World Health Organization (2010). Guidelines for the Treatment of Malaria.

[8] Zoller T, Junghanss T, Kapaun A, Gjørup I, Richter J, Hugo-Persson M (2011). Intravenous artesunate for severe malaria in travelers, Europe. Emerging Inf Dis.

[9] Kreeftmeijer-Vetger AR, Genderen PJ, Visser LG, Bierman WFW, Clerinx J, van Veldehuizen CKW (2012). Treatment outcome of intravenous artesunate in patients with severe malaria in Netherlands and Belgium. Malaria J.

[10] World Health Organization (2013). WHO Information Note on Delayed Haemolytic Anaemia Following Treatment with Artesunate.

[11] Medicine for Malaria Venture: Experts Group Meeting on Delayed Haemolytic Anaemia Following Treatment with Injectable Artesunate. Vienna, Austria, 2013. (*http:/*www.mmv.org/newsroom/events/expert-group-meeting-safety-profile-injectable-artesunate).

[12] Price RC, Simpson JA, Nosten F, Luxemberger C, Hkirjaroen L, ter Kuile F (2001). Factors contributing to anemia after uncomplicated falciparum malaria. Am J Trop Med Hyg.

[13] Sowunmi A, Gbotosho GO, Happi CT, Fateye BA (2010). Factors contributing to anaemia after uncomplicated *Plasmodium falciparum* malaria in children. Acta Trop.

[14] Ouedraogo HZ, Zeba A, Dramaix-Wilmet M, Donnen P (2008). Moderate to severe anaemia due to afebrile *Plasmodium falciparum* infections in children aged 6–23 months from rural district of Kongoussi, Burkina Faso. J Trop Pediatr.

[15] Sumbele IUN, Samje M, Nkuo-Akenji T (2013). A longitudinal study on anaemia in children with Plasmodium falciparum infection in the Mount Cameroon region: prevalence, risk factors and perceptions by caregivers. BMC Infect Dis.

[16] Biemba G, Gorduek VR, Thuma P, Weiss G (2000). Markers of inflammation in children with severe malaria. Trop Med Int Health.

[17] Helleberg M, Goka BQ, Akanmori BD, Obeng-Adjei G, Rodriques O, Kurtzhals JAL (2005). Bone marrow suppression and severe anaemia associated with persistent *Plasmodium falciparum* infection in African children with microscopically undetectable parasitaemia. Malaria J.

[18] Awah NW, Troye-Blomberg M, Berzins K, Gysin J (2009). Mechanisms of malarial anaemia: potential involvement of *Plasmodium falciparum* low molecular weight rhoptry-associated proteins. Acta Trop.

[19] Premji Z, Umeh RE, Uwusu-Agyei S, Fabian E, Ezedinachi EU, Oguche S (2009). Chlorproguanil-dapsone-artesunate versus artemether-lumefantrine: a randomized, double blind phase III trial in African children and adolescents with uncomplicated *Plasmodium falciparum* malaria. PLoS One.

[20] Gbotosho GO, Sowunmi A, Okuboyejo TM, Happi CT, Folarin OO, Michael SO (2011). Therapeutic efficacy and effect of artemether-lumefantrine and artesunate-amodiaquine cofomulated or copackaged on malaria-associated anemia in children in uncomplicated *Plasmodium falciparum* malaria in southwest Nigeria. Am J Trop Med Hyg.

[21] Oguche S, Okafor HU, Watila I, Meremikwu M, Agomo P, Ogala W (2014). Efficacy of artemisinin-based combination treatments of uncomplicated falciparum malaria in under-five year-old Nigerian children. Am J Trop Med Hyg.

[22] Gbotosho GO, Sowunmi A, Okuboyejo TM, Happi CT, Folarin OO, Adewoye EO (2011). A simple dose regimen of artesunate and amodiaquine based on age or body weight range for uncomplicated malaria in children: Comparison of therapeutic efficacy with standard dose regimen of artesunate-lumefantrine. Am J Therap.

[23] Sowunmi A, Akinrinola AA, Gbotosho GO, Okuboyejo TM, Happi CT (2012). A simple dose regimen of artesunate and amodiaquine based on arm span- or age range for childhood falciparum malaria: A preliminary evaluation. J Trop Ped.

[24] World Health Organization (2000). Severe falciparum malaria. Trans R Soc Trop Med Hyg.

[25] Sowunmi A, Balogun ST, Gbotosho GO, Happi CT (2009). Effects of amodiaquine, artesunate, and artesunate-amodiaquine of *Plasmodium falciparum* malaria-associated anaemia in children. Acta Trop.

[26] Sowunmi A, Gbotosho GO, Happi CT, Folarin O, Okuboyejo T, Michael O (2011). Use of area under the curve to evaluate the effects of antimalarial drugs on malaria-associated anaemia after treatment. Am J Therap.

[27] Rowland M, Tozer TN (1980). Clinical pharmacokinetics: concept and application.

[28] Bain BJ, Bates I, Lewis SM, Bain BJ, Bates I (2001). Basic haematological techniques. Practical Haematology.

[29] Epi Info Version 6 (1994). A Word Processing Data Base and Statistics Program for Public Health on IBM-compatible Microcomputers.

[30] SPSS for Windows Release 20.0 (standard version). SPSS Inc., Chicago IL; 2011.

[31] Bland JM, Altman DG (1986). Statistical methods for assessing agreement between two methods of clinical measurement. Lancet.

[32] Sowunmi A, Gbotosho GO, Happi C, Okuboyejo T, Folarin O, Balogun S (2009). Therapeutic efficacy and effects of artesunate-mefloquine and mefloquine alone on malaria-associated anemia in children with uncomplicated *Plasmodium falciparum* malaria in southwest Nigeria. Am J Trop Med Hyg.

[33] Fleming AF, Storey J, Molineaux L, Iroko EA, Attai ED (1979). Abnormal haemoglobins in the Sudan savanna of Nigeria. I. Prevalence of haemoglobins and relationship between sickle cell trait, malaria and survival. Ann Trop Med Parasitol.

[34] Mockenhaupt FP, Falusi AG, May J, Ademowo OG, Olumese PE, Meyer CG (1999). The contribution of α-thalassaemia to anaemia in Nigeria population exposed to intense malaria transmission. Trop Med Int Health.

[35] Bienzle U (1981). Glucose-6-phosphate dehydrogenase deficiency. Part 1: Tropical Africa. Clin Haematol.

[36] Adewunmi CO, Gebremedhin G, Becker W, Olorunmola FO, Dorfler G, Adewunmi TA (1993). Schistosomiasis and intestinal parasites in rural villages in southwest Nigeria: an indication for expanded programme on drug distribution and integrated control programme in Nigeria. Trop Med Parasitol.

[37] White NJ, Ho M, Baker JR, Muller R (1992). The pathopysiology of malaria. Adv in Parasitol 1992.

[38] Jakeman GN, Saul A, Hogarith WL, Collins WE (1999). Anaemia of acute malaria infection in non-immune patients primarily results from destruction of uninfected erythrocytes. Parasitol.

[39] Menendez C, Fleming AF, Alonso PL (2000). Malaria-related anaemia. Parasitol Today.

[40] Chotivanich K, Udomsangpetch R, Dondorp A, Williams T, Angus B, Simpson JA (2000). The mechanisms of parasite clearance after antimalarial treatment of *Plasmodium falciparum* malaria. J Infect Dis.

[41] Newton PN, Chotivanich K, Chierakul W, Ruagveerayuth R, Teerapong P, Silamut K (2001). A comparison of the in vivo kinetics of *Plasmodium falciparum* ring-infected erythrocyte surface antigen-positive and –negative erythrocytes. Blood.

[42] Angus BJ, Chotivanich K, Udomsangpetch White NJ. In vivo removal of malaria parasites from red blood cells without their destruction in acute falciparum malaria. Blood. 1997;2037–2040. 9292540

[43] Gbotosho GO, Okuboyejo TM, Happi CT, Sowunmi A (2014). Fall in haematocrit per 1000 parasites cleared from peripheral blood: a simple method for estimating drug-related fall in haematocrit after treatment of malaria infections. Am J Ther.

[44] Jauréguiberry S, Ndour PA, Roussel C, Ader F, Safeukui I, Nguyen M (2014). Postartesunate delayed hemolyssis is a predictable event related to the lifesaving effect of artemisinins. Blood.

[45] Arguin PM (2014). Case definition: postartemisinin delayed hemolysis. Blood.

[46] Rolling T, Agbenyega T, Issifou S, Adegnika J, Sylverken J, Spahlinger D (2014). Delayed hemolysis after treatment with parenteral artesunate in African children with severe malaria—a double-center prospective study. J Infect Dis.

