# Supplementary material for: Temporal changes in haematocrit following artemisinin-based combination treatments of uncomplicated falciparum malaria in children
Source: BMC Infect Dis. 2015 Oct 26;15:454. doi: 10.1186/s12879-015-1219-y (PMC4620624; doi:10.1186/s12879-015-1219-y)
Supplement: Additional file 1: Figure S1. — Haematocrit values of patients with late fall after artemisinin-based combination treatments of acute uncomplicated falciparum malaria. (DOCX 40 kb) [file 12879_2015_1219_MOESM1_ESM.docx]

**Figure S1 Haematocrit values of patients with late fall after artemisinin-based combination treatments of acute uncomplicated falciparum malaria**
