# Supplementary material for: Temporal changes in haematocrit following artemisinin-based combination treatments of uncomplicated falciparum malaria in children
Source: BMC Infect Dis. 2015 Oct 26;15:454. doi: 10.1186/s12879-015-1219-y (PMC4620624; doi:10.1186/s12879-015-1219-y)
Supplement: Additional file 2: Table T1. — Risk factors for ≥5 units fall in haematocrit from baseline in children with uncomplicated falciparum malaria treated with artesunate-amodiaquine or artemether-lumefantrine. (DOCX 12 kb) [file 12879_2015_1219_MOESM2_ESM.docx]

**Table T1 Risk factors for ≥5 units fall in haematocrit from baseline in children with uncomplicated falciparum malaria treated with artesunate-amodiaquine or artemether-lumefantrine**

| **Variable** | **Total no.** | **No. with late anaemia*** | **OR (95% CI)** | **P value** |
| --- | --- | --- | --- | --- |
| **Gender**  Male  Female | 131  117 | 27  24 | 1  1.0(0.5-1.9) | 0.89 |
| **Age (years)**  <6  ≥6 | 126  122 | 20  31 | 1  1.8 (0.3 –3.4) | 0.09 |
| **Duration of illness (days)**  >2 days  ≤2days | 142  106 | 29  22 | 1  1.0(0.5-1.8) | 0.9 |
| **Enrolment body temperature**  ≥37.4^o^C  <37.4^o^C | 199  49 | 40  11 | 1  0.9 (0.4–1.8) | 0.88 |
| ≥40^o^C  <40^o^C | 16  232 | 3  48 | 1  0.9(0.2-3.2) | 0.89 |
| **Parasitaemia (µL^-1^)**  <100,000  ≥100,000 | 177  71 | 28  22 | 1  2.4 (1.3 – 4.6) | 0.012 |
| **Total doses of artesunate**  >10mg/kg  ≤10mg/kg | 120  51 | 28  17 | 1  0.7 (0.4 – 1.5) | 0.48 |

* haematocrit <30%
