# Supplementary material for: Temporal changes in haematocrit following artemisinin-based combination treatments of uncomplicated falciparum malaria in children
Source: BMC Infect Dis. 2015 Oct 26;15:454. doi: 10.1186/s12879-015-1219-y (PMC4620624; doi:10.1186/s12879-015-1219-y)
Supplement: Additional file 3: Figure S2. — Semilog plots of deficit in haematocrit from 30 % versus time in children with early monophasic fall (Pattern 2) [A] or late monophasic fall in haematocrit (Pattern 3) [B] following artemisinin-based combination treatments of uncomplicated falciparum infections. (DOCX 24 kb) [file 12879_2015_1219_MOESM3_ESM.docx]

**A**

**0**

**7**

**14**

**21**

**28**

**0.1**

**1**

**10**

**Time (days)**

**Deficit in haematocrit (%)**

**0**

**7**

**0.1**

**1**

**10**

**14**

**21**

**28**

**35**

**42**

**49**

**Time (days)**

**Deficit in haematocrit (%)**

**B**

**Figure S2 Semilog plots of deficit in haematocrit from 30% versus time in children with early monophasic fall (Pattern 2) [A] or late monophasic fall in haematocrit (Pattern 3) [B] following artemisinin-based combination treatments of uncomplicated falciparum infections**

**B**
