# Supplementary material for: Temporal changes in haematocrit following artemisinin-based combination treatments of uncomplicated falciparum malaria in children
Source: BMC Infect Dis. 2015 Oct 26;15:454. doi: 10.1186/s12879-015-1219-y (PMC4620624; doi:10.1186/s12879-015-1219-y)
Supplement: Additional file 4: Figure S3. — Semilog plots of deficit in haematocrit from 30 % versus time in children with haematocrit <30 % at presentation (Pattern 6). (DOCX 16 kb) [file 12879_2015_1219_MOESM4_ESM.docx]

**0**

**7**

**14**

**21**

**28**

**0.1**

**1**

**10**

**Deficit in haematocrit (%)**

**Figure S3 Semilog plots of deficit in haematocrit from 30% versus time in children with haematocrit <30% at presentation (Pattern 6)**
