# Supplementary material for: Temporal changes in haematocrit following artemisinin-based combination treatments of uncomplicated falciparum malaria in children
Source: BMC Infect Dis. 2015 Oct 26;15:454. doi: 10.1186/s12879-015-1219-y (PMC4620624; doi:10.1186/s12879-015-1219-y)
Supplement: Additional file 5: Figure S4. — Scatter plots of half-time of the decline in haematocrit deficit from 30 % and anaemia recovery time children who were anaemic at presentation (r = 0.55, P < 0.0001). (DOCX 36 kb) [file 12879_2015_1219_MOESM5_ESM.docx]

**Figure S4 Scatter plots of half-time of the decline in haematocrit deficit from 30% and anaemia recovery time children who were anaemic at presentation (r = 0.55, P < 0.0001)**
